# Mapping Therapeutic Regulatory T Cell Fate with MRI: Current Strategies and Translational Outlook

**DOI:** 10.3390/nano16110691

**Published:** 2026-06-01

**Authors:** Yu Ping, Lydia Chen, Jacob Joel Hoenig, Xiaohan Yang, Fanny Chapelin

**Affiliations:** 1 Shu Chien-Gene Lay Department of Bioengineering, University of California San Diego, La Jolla, CA 92093, USA; 2 Department of Radiology, University of California San Diego, La Jolla, CA 92093, USA

**Keywords:** regulatory T cells, adoptive cell therapy, MRI cell tracking, superparamagnetic iron oxide, fluorine-19 MRI, transplantation, graft-versus-host disease

## Abstract

Adoptive cell therapies, and more specifically, regulatory T cell (Treg) therapies, have shown significant therapeutic promise across multiple immune-mediated diseases including graft-versus-host disease (GvHD), solid organ transplant (SOT) rejection, and autoimmune diseases. One key challenge is the lack of insight into the biodistribution and fate of adoptively transferred T cells and Tregs in living organisms. These uncertainties delay progress on establishing optimal dosage(s), infusion timing and route, as well as investigations into off-target effects. Magnetic resonance imaging (MRI) cell tracking is particularly beneficial in this setting because it enables real-time, deep-tissue coverage without ionizing radiation. In this review, we compare existing MRI T cell tracking strategies using iron oxide particles and fluorinated agents. We describe preclinical and clinical applications of MRI for cell therapy tracking and provide a perspective on the potential impact on the field.

## 1. Introduction

Immune tolerance is essential for health, yet it can fail in several severe clinical situations. Graft-versus-host disease (GvHD), solid organ transplant (SOT) rejection, and autoimmune diseases occur when adaptive immune responses get misdirected, causing immune cells to attack tissues they should otherwise protect. While these conditions present differently and occur in various contexts, they all stem from a common failure in T cell tolerance, characterized by unchecked effector T cell activity and insufficient Treg control. In GvHD, donor-derived T cells attack host tissues following allogeneic hematopoietic stem cell transplantation, which leads to tissue damage and substantial treatment-related mortality [1]. Occasionally, similar events can also occur after solid organ transplantation or blood transfusion [2]. SOT rejection represents an opposite scenario: host immune cells recognize donor alloantigens and gradually destroy the graft. Ultimately, graft function is compromised, leading to late graft failure despite modern immunosuppressive therapy [3]. Autoimmune diseases represent yet another variant of this issue, where autoreactive lymphocytes target self antigens due to failures in central and peripheral tolerance mechanisms [4]. Together, GvHD, transplant rejection, and autoimmunity illustrate clinically distinct conditions that share a common biological issue: insufficient immune tolerance [4]. Achieving immune tolerance requires a finely tuned balance between Tregs and effector T cells, a balance that, when disrupted, can tip the immune system from protective to harmful.

At the heart of immune tolerance are CD4^+^CD25^+^FOXP3^+^ Tregs, a specialized subset of CD4^+^ T cells marked by FOXP3 expression [5]. Tregs play a critical role in limiting excessive immune activation by restraining both autoreactive and alloreactive T cells, and by maintaining tissue homeostasis [4]. Issues with Treg numbers, stability, or function are linked to more severe GvHD, reduced transplant tolerance, and many autoimmune conditions [6]. As a result, numerous researchers now consider GvHD, SOT rejection, and autoimmunity as interconnected forms of “failure of immune tolerance” that could benefit from strategies to restore Treg activity [7].

Adoptive cell therapies (ACTs) using natural or genetically modified Tregs, including polyclonal Treg transfer, antigen-specific Tregs, and CAR-Tregs [4], have shown significant promise as immune-based treatments. They have been validated as a means to promote organ tolerance and reduce long-term reliance on broad immunosuppression in GvHD [8,9], SOT, and autoimmune diseases. In these scenarios, Treg therapy is seen as a way to reshape immune responses, rather than just suppressing them [10]. However, translating adoptive Treg transfer into clinical practice faces several challenges. Evaluations of Treg therapy indicate uncertainty about in vivo persistence, tissue homing, and dose–response relationships, as well as concerns about phenotypic instability and decline in suppressive function over time [11]. In this review, we use “Treg fate” to encompass two related but distinct definitions: biodistributional fate, defined as where transferred cells traffic, accumulate, persist, or clear over time, and functional fate, defined as whether those cells remain viable, phenotypically stable, and immunosuppressive after transfer. Research in transplantation and autoimmunity also points out that Tregs may act at bystander sites, potentially affecting non-target immune pathways. This raises concerns about off-target effects or unintended systemic immunosuppression [3]. Conventional clinical readouts using peripheral blood counts or serum biomarkers only provide indirect information about where transferred Tregs go and how long they remain functional [1]. These gaps in knowledge make it difficult to optimize cell dose, timing, and route of administration for Treg therapy in major disease settings [12].

Non-invasive imaging of adoptively transferred T cells offers a way to address these questions. Among whole-body imaging modalities, magnetic resonance imaging (MRI) has shown that non-invasive tracking can reveal T cell fate such as migration paths, accumulation in target organs, and clearance over time in both preclinical and clinical settings [13,14]. Methods utilizing SPION, ^19^F perfluorocarbon nanoemulsions, gadolinium agents, and reporter gene systems allow for longitudinal MR imaging without repeated invasive biopsies [15]. These methods can address critical questions currently facing clinicians and researchers: whether Tregs can reach relevant organs or microenvironments; what the time window for peak accumulation is; how cell dose and survival duration correlate with efficacy or toxicity; whether imaging readings can aid in developing personalized dosing regimens or adaptive treatment plans [13].

This review aims to address these concerns by integrating four key elements: the role of T cells and Tregs in GvHD, SOT rejection, and autoimmune disease; the current status of adoptive Treg transfer; the available in vivo imaging methods for Treg tracking with an emphasis on MRI; a structured comparison of MRI labeling methods in terms of sensitivity, resolution, safety, and feasibility for clinical translation. Understanding how MRI has been used to image T cells and Tregs in vivo thus far, and what technical constraints still hinder clinical translation, is the central goal of this review.

## 2. Role of T Cells and Tregs in Immune Tolerance

### 2.1. Immune Recognition and Tolerance Failure in Transplantation

When the immune system detects non-self antigens in transplanted tissue, it can trigger an allogeneic immune response that results in graft rejection or GvHD. In transplantation and autoimmune disorders, immune tolerance means that the body does not recognize specific antigens as foreign. This prevents harmful immune attacks while allowing normal immune defense to function.

Differences in antigens between the donor and recipient form the basis for allogeneic rejection. Major histocompatibility complex (MHC) antigens are the most variable between individuals, while additional minor histocompatibility antigens (mHAs) can also contribute to an immune reaction [16]. However, the inflammatory environment during transplantation amplifies such response. Ischemia–reperfusion injury creates cellular stress and tissue damage, which leads to the release of damage-associated molecular patterns (DAMPs) that activate innate immune pathways [17]. Pre-transplant conditioning, such as chemotherapy and radiation, can worsen this situation by disrupting mucosal barriers and allowing microbial products with pathogen-associated molecular pattern (PAMP) activity to enter the bloodstream [18]. Together, these signals activate antigen-presenting cells (APCs) [19]. This promotes their maturation, cytokine release, and co-stimulatory signaling, all of which improve T cell priming [19].

**Figure 1:**
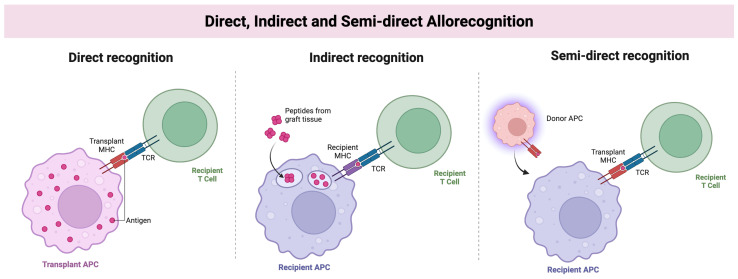
T cell allorecognition pathways: direct, indirect, and semi-direct. In the direct pathway, transplant APCs interact directly with recipient T cells. In indirect recognition, recipient APCs present processed transplant peptides (alloantigens) to recipient T cells. In the semi-direct pathway, recipient APCs acquire transplant HLAs that present peptides directly to recipient T cells. APCs: antigen-presenting cells; TCR: T cell receptor. Created in BioRender. Ping, Y. (2026) https://BioRender.com/9r755y5.

In transplantation, recipient T cells recognize donor antigens through three main pathways (Figure 1). In the direct pathway, donor APCs are transferred with the graft and directly present donor MHC molecules to host T cells [19]. In the semi-direct pathway, host APCs obtain intact donor MHC-peptide complexes from donor cells. In the indirect pathway, host APCs process donor-derived antigens and present them on self-MHC molecules (Figure 1). In all cases, T cells then recognize these peptide antigens presented by MHC molecules on APCs using their T cell receptor (TCR). Two other signals are required for T cell activation, including co-stimulatory molecules, like CD28–CD80/86 interactions, and cytokines such as IL-2 [18].

Once activated, T cells divide into effector and memory populations that contribute to graft injury [20]. CD8^+^ cytotoxic T cells cause target-cell death through perforin/granzyme and Fas-FasL pathways [21]. Meanwhile, CD4^+^ helper T cells enhance immune responses by activating APCs and supporting B-cell antibody production [22]. Pre-existing donor-reactive memory T cells speed up rejection due to their quick responsiveness and increased cytokine production [23]. Altogether, these pathways cause tissue damage and lead to both cellular and antibody-mediated rejection [20].

Immune tolerance is maintained through both central and peripheral mechanisms. Central tolerance takes place in the thymus, where strongly self-reactive T cell clones are removed during development [24]. Peripheral tolerance targets cells that escape thymic selection and includes deletion, functional silencing, and active immune suppression [25]. Among these regulatory processes, Tregs play a crucial role in maintaining immune balance. CD4^+^CD25^+^FOXP3^+^ Tregs are a specialized subset of CD4^+^ T cells identified by FOXP3 expression and high levels of the IL-2 receptor alpha chain CD25 [26]. They suppress immune responses through various complementary mechanisms, like releasing inhibitory cytokines such as IL-10 and TGF-β, consuming local IL-2, modulating co-stimulatory signaling, and suppressing antigen-presenting cell activity [26]. Through these actions, Tregs limit excessive effector T cell activation and help maintain tissue tolerance.

The role of Tregs is well-known in both alloimmune and autoimmune contexts. In GvHD, a decrease in Tregs worsens disease severity, while the transfer of Tregs reduces alloreactive T cell expansion and limits tissue damage [9]. Similarly, issues with Treg number or function have been linked to autoimmune diseases like type 1 diabetes, systemic lupus erythematosus, and multiple sclerosis [27]. During transplantation, GvHD, and autoimmunity, the balance between regulatory and effector T cells is a key factor in determining immune outcomes. When this balance is disrupted, harmful immune responses can arise [28]. When it is restored, immune tolerance can be re-established. This idea underpins Treg-based therapies aimed at restoring balanced immunity.

### 2.2. T Cells and Tregs as Therapeutic Agents

The complex roles of T cells in immune activation and regulation make them an appealing treatment option. Effector T cells help destroy infected and cancerous cells [29]. In contrast, Tregs maintain immune balance by controlling excessive or misguided immune responses [26]. Their ability to be expanded [30,31], genetically modified, and functionally changed outside the body has made T cells highly flexible as cellular treatments [32,33]. This idea forms the basis of adoptive T cell therapy (ACT). In ACT, T cells are isolated, altered outside the body, and then reinfused to target immune responses more precisely and effectively [34]. In the last ten years, ACT has developed in two main directions: improving immunity through effector T cell therapies against cancer [35], and restoring tolerance through regulatory T cell therapies [36,37]. Effector ACT includes tumor-infiltrating lymphocyte (TIL) therapy, TCR-engineered T cells, and chimeric antigen receptor (CAR)-T cells. Each has played an important role in advancing cancer immunotherapy [38,39,40].

**Figure 2:**
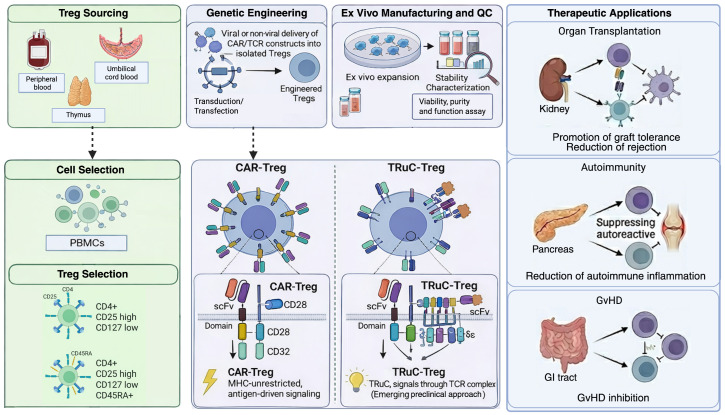
Engineered Treg development and therapeutic use workflow.Tregs can be collected from peripheral blood, umbilical cord blood, or the thymus. In human studies, these cells are usually isolated as CD4^+^CD25^high^CD127^low^ cells. A more naïve Treg population can also be enriched by selecting CD45RA^+^ cells. After isolation, the cells can be engineered to express antigen-directed receptor constructs. These designs can include CARs or TRuCs. Viral and non-viral methods can be used for this step. The engineered cells are then expanded and tested before use. Testing steps often include checks for cell phenotype, viability, purity, and suppressive function. The engineered Treg products, including CAR-Tregs and TRuC-Tregs, can be used in transplantation or autoimmune disease and GvHD treatment. In these settings, the goal is to limit harmful immune activity and support immune tolerance. Abbreviations: PBMCs, peripheral blood mononuclear cells; CAR, chimeric antigen receptor; TRuC, T cell receptor fusion construct; GvHD, graft-versus-host disease; QC, quality control; GI, gastrointestinal. Created in BioRender. Ping, Y. (2026), https://BioRender.com/ojl25nz.

In parallel, Treg-based therapies have shown promise in reducing harmful immunity and restoring immune tolerance. Current methods involve modifying natural Tregs with drugs [36,41], and the adoptive transfer of expanded or engineered Tregs outside the body [36,42] (Figure 2). Among adoptive methods, polyclonal Tregs provide broad immunosuppression [36]. Antigen-specific Tregs allow for more targeted immune control [43], while CAR-Tregs provide enhanced precision through engineered antigen recognition [42,44,45]. Among these, CAR-Tregs are one of the fastest-growing approaches [42]. By allowing MHC-independent antigen recognition, CARs work around the challenges that come with standard antigen presentation and HLA restrictions [44,45]. This makes them especially appealing for transplantation and autoimmune treatments [46]. However, their therapeutic effect relies not just on targeting antigens but also on maintaining the regulatory phenotype. CAR signaling strongly affects FOXP3 stability and suppressive ability [47,48,49]. Accordingly, there remains a growing need for simple, non-invasive approaches to assess Treg stability, suppressive function, and persistence following administration in vivo.

In recent years, the clinical application of Treg ACT has sped up significantly, with over 260 registered trials testing these therapies in transplantation, GvHD, and autoimmune diseases [10,42,50]. Early research has shown encouraging evidence that these therapies can promote immune tolerance and reduce harmful immune responses [10,37]. Despite this progress, wider use is still limited by a lack of understanding of Treg behavior after infusion [10,42]. This is especially true with regard to how they traffic, persist, and remain stable in living organisms. Notably, analyses of peripheral blood only offer limited insight into these processes [10]. Effective immunoregulation depends on the coordinated expansion of Tregs within lymphoid tissues, followed by their movement to sites of inflammation [51]. These obstacles highlight the increasing need for non-invasive methods to monitor Treg status in living organisms [10].

## 3. In Vivo Imaging Modalities and the Rationale for MRI-Based Cell Tracking

### 3.1. Imaging Approaches for Tracking Therapeutic T Cells In Vivo

A variety of methods can be used to track T cells in vivo (Table 1). Optical imaging methods offer the advantages of single cell resolution and multi-scale visualization [52,53]. However, their limited tissue depth penetration, depth-dependent signal attenuation, and toxicity associated with imaging probes largely restrict them to preclinical applications only. Ultrasound is cost-effective, radiation-free, and has deep penetration and real-time imaging capability [54,55]. Nonetheless, it lacks cell tracking contrast agents. Photoacoustic imaging (PAI) offers a high contrast-to-noise ratio and high-resolution images with deep penetration, but also falls short with regard to contrast agents for immune cell tracking [56]. Positron Emission Tomography (PET) and Single Photon Emission Computed Tomography (SPECT) offer the highest sensitivity for cell tracking and can be applied clinically. Reporter-based strategies using engineered T cells expressing herpes simplex virus thymidine kinase (HSV-TK) or prostate-specific membrane antigen (PSMA) have been used to image CAR-T cell trafficking in clinical studies [57,58,59]. However, PET and SPECT require the use of ionizing radiation for image acquisition, have limited spatial resolution compared to MRI, and the short half-lives of many radiotracers constrain longitudinal monitoring.

MRI offers several properties that make it well-suited for longitudinal immune cell tracking: high spatial resolution, unlimited depth penetration, 3D whole-body coverage, the ability to acquire multiple timepoints from a single experiment, and the absence of ionizing radiation [15,74]. These features are particularly relevant for Treg therapy to provide a direct readout of biodistributional fate, where the key biological questions such as whether infused cells reach target tissues, how long they persist, and whether they remain in relevant compartments, require repeated imaging over days to weeks rather than a single snapshot. Although specific contrast agents’ constraints will be explored further in Section 3, broad MRI challenges should be given consideration. Live labeled cells cannot be reliably separated from dead cells that retain their label, nor from host phagocytes that have scavenged released particles. Direct labels also dilute with cell division, limiting the window for longitudinal tracking.

Magnetic particle imaging (MPI) is an emerging complement to MRI that directly detects Superparamagnetic Iron Oxide (SPIO) magnetization rather than relying on SPIO-induced changes in proton signal, yielding zero tissue background and linear cell quantification. Preclinical detection limits have reached as low as 250–1000 labeled cells [75], making its sensitivity competitive with nuclear imaging while avoiding ionizing radiation. MPI and MRI are naturally paired; MPI provides sensitive, quantitative cell detection, whereas MRI provides high-resolution anatomical localization, but practical barriers persist. MRI spatial resolution degrades at the human-bore scale, and MPI has not yet established a FDA-approved platform for scanner or tracer cell tracking. Additionally, dual MRI/MPI systems are not yet available. For Treg applications, it is best understood as a promising future complement to MRI rather than a currently deployable translational tool.

Future MRI-based Treg tracking studies should nonetheless pair imaging with orthogonal biological assays to assess the functional fate of infused Tregs [76]. In transplantation applications, Halloran et al. and Bloom et al. showed that liquid biopsy approaches such as donor-derived cell-free (dd-cf) DNA assay may help assess graft injury [77,78]. However, increased dd-cfDNA reflects graft cell death and correlates with allograft rejection, but is not specific for rejection or Treg function [77,78]. Multimodal imaging can further connect localization with limited functional context. For example, a combined PET/MR system could provide a more comprehensive picture of the transplant microenvironment. MRI would provide high-resolution anatomical localization, while PET reporter systems such as HSV1-tk [57] or PSMA [59] could provide a viability-linked and longitudinal whole-body tracking of engineered, reporter-expressing T cells. In GvHD, Bioluminescence Imaging (BLI) could also provide viable Treg cell persistence or expansion information based on firefly luciferase readouts [51]. However, BLI is mainly suited for the preclinical space because of its optical photon attenuation, scattering, and depth-dependent signal loss which limits its use for deep-tissue cell tracking [79].

A notable multimodal example specific to T cell therapy comes from Kiru et al., who combined ferumoxytol-labeled CAR-T cell tracking with MRI, photoacoustic tomography (PAT), and magnetic particle imaging (MPI) in an osteosarcoma mouse model [80]. For Treg tracking in transplantation, multimodal platforms could be valuable. Dual-modal fluorine MRI/fluorescence nanoemulsions have also been reported, enabling both MRI detection and post-mortem fluorescence histological correlation [81]. The ongoing challenge is integrating multimodal labels into good manufacturing practice (GMP)-compliant manufacturing without compromising cell viability or function.

Taken together, no existing modality matches MRI’s combination of soft-tissue resolution, depth penetration, longitudinal capability, and clinical infrastructure for tracking adoptively transferred T cells and Tregs in vivo. PET and SPECT provide much higher cell detection sensitivity than MRI, and reporter gene strategies allow for repeated imaging over weeks without diluting the label. However, sensitivity is not the main challenge for monitoring Treg therapy. The key clinical question is not whether transferred cells are somewhere in the body, but whether they reach and gather in the target organ, such as the transplanted kidney, the GvHD-affected gut, or the inflamed CNS, and whether that gathering relates to therapeutic response. To answer that question, we need the soft-tissue anatomical context and spatial co-registration that PET and SPECT, with their centimeter-scale resolution, cannot reliably give. MRI’s millimeter resolution, in the same imaging session that pinpoints the cell signal, makes it perfectly suited for this biological question. Optical imaging provides single-cell resolution but is limited to preclinical use due to depth issues that prevent clinical application. MPI is well-suited for detecting SPION-labeled cells and may eventually work alongside MRI for quantitative readings, but the lack of human-scale scanners and approved tracers keeps it out of current clinical reach. Overall, MRI is not just a backup choice; it is the method that best meets the specific needs of Treg therapy monitoring as the field progresses to human studies.

### 3.2. MRI Contrast Agents: Superparamagnetic Iron Oxide Nanoparticles (SPIONs)

The concept of using iron oxide particles to specifically label lymphocytes for MRI dates to work by Bulte and colleagues in 1992, who repurposed dextran-magnetite particles as MRI contrast agents [82]. By coupling these particles to anti-lymphocyte monoclonal antibodies via a biotin-streptavidin bridge, human lymphocytes could be selectively labeled, producing strong and selective negative contrast enhancement of lymphocyte suspensions at 2.0 T. SPION remain the most established MRI labels for T cell tracking [14,83,84,85]. These particles create local magnetic field disturbances that appear as signal loss on T2-weighted and T2*-weighted images [86]. This effect can be highly sensitive because the signal void extends beyond the physical size of the particle, which produces the well-known “blooming” artifact. This sensitivity helped make iron oxides the earliest and most widely used MRI cell tracking platform.

**Figure 3:**
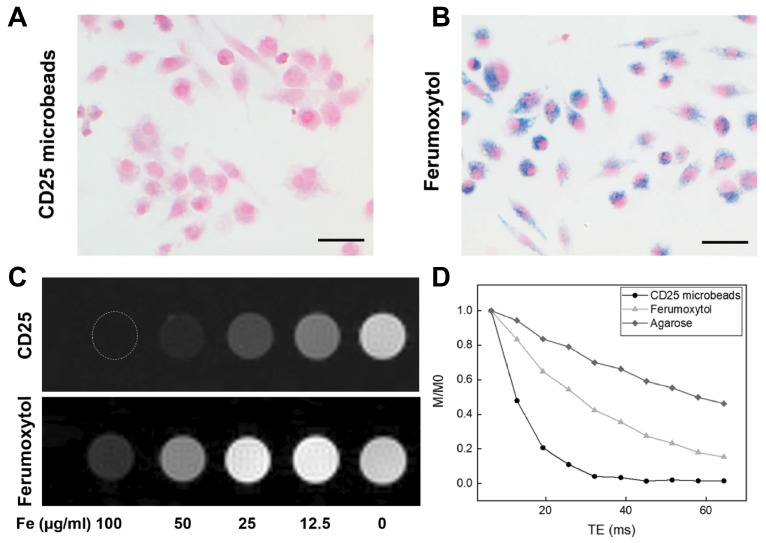
CD25-SPIONs show limited uptake by unspecific phagocytic cells but generate stronger MR contrast than ferumoxytol. (**A**) Prussian blue iron staining shows minimal intracellular iron staining in macrophages exposed to CD25-SPIONs, while (**B**) ferumoxytol produces abundant intracellular blue deposits. Scale bar represents 100 µm. (**C**) T2-weighted agarose phantoms show stronger signal decay with CD25-SPIONs than clinical standards, ferumoxytol at equivalent iron concentrations. (**D**) Corresponding T2 relaxation curves show strongest effect of the CD25-SPIONs compared to ferumoxytol. Linear agarose gel decay is displayed as reference. Adapted from [91] under a Creative Commons Attribution 4.0 International License.

The main practical problem is that T cells are not naturally phagocytic, so they do not take up extracellular particles well by simple incubation. Because of this, many studies have relied on transfection agents, electroporation, or redesigned particles to improve intracellular loading [80,87,88,89,90]. Garden et al. developed an early solution by conjugating the HIV-tat cell-penetrating peptide to ultrasmall SPIONs (USPIOs), enabling efficient labeling of CD4^+^ T cells within 5 min while preserving their proliferative, regulatory, and migratory behavior in vitro [89]. Liu et al. subsequently reported PEG-coated iron oxide nanoparticles that labeled rat and human T cells at high efficiency without transfection agents. Kiru et al. demonstrated the feasibility of labeling CAR-T cells with the FDA-approved iron supplement ferumoxytol (a commonly used SPION) using a microfluidics-based mechanoporation device, achieving significant nanoparticle uptake while preserving T cell proliferation, viability, and function [80]. Another success in MRI-based T cell tracking was demonstrated by Jin et al., where MIRB (Molday Ion Rhodamine-B)-labeled CD4^+^ T cells were visualized in the mouse brain and peripheral organs, providing a model to track CD4^+^ T cells in ischemic brain injury. Khurana et al. showed that CD25 magnetic sorting microbeads, which are already used to isolate Tregs, can also serve as MRI labels and produce stronger intracellular labeling than ferumoxytol in that setting (Figure 3). More recently, CD4-SPION labeling was used to track systemically injected CD4^+^ T cells longitudinally in mice, with detectable signal persisting to 72 h [85]. For transplantation studies, this type of workflow is appealing because the same sorting step used to enrich Tregs can also produce MRI-visible cells.

Additionally, iron oxides still come with important limitations. These directly limit the experimental questions that iron oxide MRI can reliably answer in the context of Treg therapy. In an inflammatory transplant microenvironment, which is where Treg tracking is most clinically relevant, hemorrhage, microbleeds, air–tissue interfaces, and endogenous iron all cause T2* signal loss that looks the same as labeled cells, complicating interpretation [92,93]. Additionally, MRI cannot reliably separate live transferred T cells from dead labeled cells or from host phagocytes that have taken up released particles, and the label becomes diluted as cells divide [92,94]. This is not a minor technical issue; it means that SPION-based imaging in inflamed allografts or GvHD target organs cannot reliably link hypointense signals to viable infused Tregs on its own. Therefore, we find that iron oxide MRI is best used as a tool for early biodistribution and short-term homing studies in pre-inflammatory or controlled preclinical settings. It is not suited for long-term Treg fate monitoring in active immune-mediated disease. For that application, the specificity issues with SPION support the use of ^19^F-based methods, which we will discuss below, despite their sensitivity drawbacks.

### 3.3. MRI Contrast Agents: Fluorine-19 (^19^F) Emulsions

Fluorinated agents have shown a similarly favorable functional profile in T cell studies, with the added advantage of background-free detection. Most tissues contain essentially no detectable mobile ^19^F signal, so labeled cells appear as hotspots with minimal background [95]. In practice, investigators acquire a ^19^F image to localize the labeled cells, and a conventional ^1^H image to show subject anatomy, and then overlay the two datasets. This platform was established in early work from Ahrens and colleagues [95] and became a defining feature of perfluorocarbon-based immune cell imaging. Common labeling agents are perfluorocarbon nanoemulsions composed of perfluoropolyether (PFPE) or perfluoro-15-crown-5-ether (PFCE) microfluidized in a block co-polymer (F68) or lipid shell (e.g., phospholipid) [96,97,98,99,100,101].

Another major strength of ^19^F MRI is quantification. Since the signal scales with the amount of fluorine present, it can be related to apparent cell number when the fluorine load per cell and a reference standard are known [102]. This platform has been extensively developed for macrophage [98,101,103], dendritic cell [104,105] and T cell [100,104,106] tracking in the context of cancer. For adoptive T cell and Treg studies, this quantitative feature is especially useful because dose, homing, and persistence are central determinants of therapeutic outcome.

The major weakness of ^19^F MRI is sensitivity. The minimum detectable burden is often above 10^6^ cells per voxel, depending on scanner field strength, pulse sequence, and cell type. Treg therapy trials typically infuse 10^8^ to 10^9^ cells distributed body-wide [107], meaning that organ-level accumulation will be detectable only where cells concentrate sufficiently—a condition that may apply to the target organ, but is unlikely to be met diffusely. This sensitivity gap is currently the single largest barrier to clinical ^19^F Treg tracking, and it is an engineering problem, not a fundamental physical constraint. Highly concentrated fluorine nanoemulsion formulations [81,108], improved ^19^F-tuned radiofrequency coils [109], and noise-robust pulse sequences optimized for low signal-to-noise environments [110,111] are all active areas of development that could meaningfully close this gap within the next few years. The method also depends on careful co-registration between the ^19^F and ^1^H datasets, so motion or misalignment can place the hotspot in the wrong anatomical location. Like other direct labels, fluorine is diluted by cell division and may persist after cell death if the label is transferred to phagocytes. The sensitivity trade-off of ^19^F MRI is real but addressable; the specificity problems of SPION in active inflammation are more resistant to engineering solutions. Overall, ^19^F MRI offers much cleaner specificity and better quantification than iron oxides for T cell tracking, but this comes at the cost of weaker sensitivity and heavier hardware demands.

### 3.4. T Cell Labeling: Functional Integrity and Manufacturing Considerations

A prerequisite for any cell labeling strategy intended for clinical use is that it does not compromise the biological properties of the labeled cells [76]. For Tregs specifically, this means preserving FOXP3 expression, suppressive function, and migratory capacity after labeling. Several studies have addressed this question for iron oxide and ^19^F agents. Garden et al. showed that USPIO labeling via a cell-penetrating peptide did not impair CD4+ T cell proliferation or migratory behavior in vitro [89]. Gedaly et al. and Ping et al. confirmed that CD25 and CD4 microbead labeling preserved Treg viability and suppressive capacity as assessed by flow cytometry [85,112]. For ^19^F labeling, Srinivas et al. demonstrated that perfluorocarbon-labeled CD4^+^ T cells retained normal migration and proliferative function in the NOD diabetes model [104], while Chapelin et al. showed that PFC-labeled CAR-T cells maintained cytotoxic function and tumor homing in vivo [100]. Nonetheless, systematic functional validation across Treg subtypes, including CAR-Tregs and antigen-specific Tregs, and across labeling platforms, remains incomplete. More critically, no published study to date has specifically assessed whether labeling affects FOXP3 stability or the Treg/effector conversion ratio under inflammatory conditions. We consider this a crucial unresolved question in the field, and a prerequisite rather than an optional validation for any IND-enabling Treg tracking study. The reason is straightforward: an imaging study that accurately maps Treg biodistribution but fails to account for the possibility that the labeling procedure itself destabilizes FOXP3 and drives phenotypic conversion under the inflammatory milieu of a rejecting graft or active GvHD cannot be interpreted as evidence of therapeutic Treg activity. Demonstrating labeling compatibility with in vitro assays under standard conditions, as the current literature predominantly provides, is necessary but insufficient. What is needed are in vivo functional readouts [76], ideally co-tracking FOXP3 reporter expression alongside MRI signal in established inflammatory models, before MRI-labeled Treg products can be considered analytically validated for clinical use.

Another practical challenge in translating MRI cell tracking to Treg therapy is label dilution during ex vivo expansion. Clinical protocols typically involve 1000- to 10,000-fold expansion over two to four weeks, meaning a label loaded at the start of manufacturing will be progressively diluted with each cell division. For iron oxide particles, it is estimated that SPION signal per cell falls below MRI detectability within days of active proliferation [13]; the same dilution kinetics apply to ^19^F agents [100]. The practical solution would be to label cells immediately before infusion rather than at the start of expansion. This strategy has been validated for ^19^F agents in a clinical-scale workflow by O’Hanlon et al., but introduces an additional GMP-regulated step that must not disturb final release criteria for Treg phenotype and suppressive function [113]. Addressing these questions prospectively rather than as an afterthought will be essential for any MRI Treg tracking study with a credible path to clinical translation.

## 4. MR Treg Tracking in the Preclinical Space

### 4.1. Solid Organ Transplantation and Rationale for Graft-Versus-Host Disease

**Figure 4:**
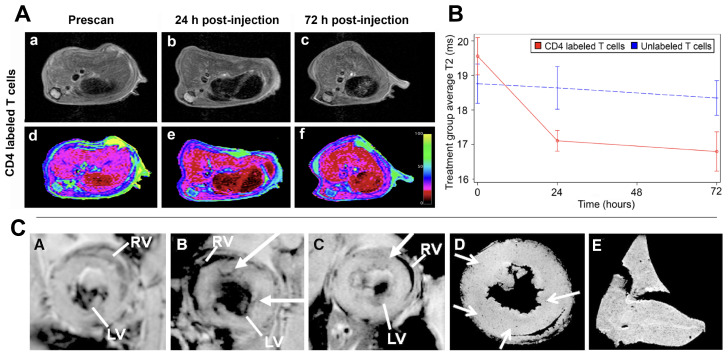
Longitudinal in vivo tracking of SPION-labeled T cells in liver, heart and lung tissues. (**A**) Representative liver MRI and corresponding T2 color maps at (**a**,**d**) prescan, (**b**,**e**) 24 h, and (**c**,**f**) 72 h after intravenous injection show persistent T2 shortening in mice receiving labeled cells (liver magenta signal turning deep red upon infiltration). (**B**) Quantitative analysis confirms significant liver T2 reduction in labeled cell recipients at 24 h, persisting through 72 h. Adapted from Ref. [85] under a Creative Commons Attribution 4.0 International License. (**C**) Representative T2*-weighted in vivo MR images and ex vivo magnetic resonance microscopy images of transplanted heart and lung demonstrate post-infusion hypointense signal in the grafts. Heart images are shown (**CA**) before infusion, (**CB**) 24 h, (**CC**) 48 h, and (**CD**) ex vivo on post-operational day 6; (**CE**) lung allografts are also shown. White arrows indicate regions of signal loss consistent with labeled T cell accumulation. Adapted with permission from Ref. [118]. Copyright © 2012 Elsevier Inc.

Most MRI cell tracking work in transplantation has measured immune infiltration or rejection biology [114,115,116,117], and those studies often focus on innate cells such as macrophages rather than on infused Tregs. Several classic transplantation MRI studies established that iron-oxide-based labeling can report immune cell accumulation in rejecting grafts as signal loss on T2*-weighted images. Liu et al. developed superparamagnetic nano-sized iron oxide particle (IOPC-NH2) labeling to track T cells by MRI in a rat transplantation model, demonstrating that labeled cells could be detected at sites of rejection, though the focus remained on effector rather than regulatory populations (Figure 4) [118]. Hitchens et al. used ^19^F MRI and spectroscopy in rat heart and kidney transplant rejection models after in vivo perfluorocarbon labeling of monocytes/macrophages, contrasting ^19^F readouts with T2*-weighted approaches and noting that hypointense regions can arise from rejection-related hemorrhage, which can complicate iron oxide interpretation [119]. In a mouse heart graft model, Flögel et al. likewise reported ^1^H/^19^F MRI detection of early rejection through a macrophage host response after intravenous perfluorocarbon administration, with signal in allografts and not in isografts [120]. These studies establish realistic organ targets, time scales, and confounds that also shape any attempt to track infused Tregs in vivo. Nonetheless, pro-inflammatory and anti-inflammatory events imaging represent distinct experimental paradigms. One asks how the host mounts a destructive response; the other asks where the therapeutic cell population goes.

A key requirement for MRI Treg tracking is a labeling strategy that is compatible with Treg biology and with clinical Treg isolation and expansion workflows. Khurana et al. proposed that magnetic cell-sorting beads, which many laboratories already use for immune cell isolation, can also serve as a MRI contrast agent [91]. They tested CD25 microbeads as a labeling method for Tregs and reported that labeled cells were detectable by MRI after in vivo delivery, including a liver signal readout in mice. Ping et al. further advanced this strategy by developing a receptor-mediated SPION labeling approach for CD4^+^ T cells aimed at longitudinal MRI tracking after systemic injection (Figure 4) [85]. In a more recent study of bilateral skin grafts comparing allogeneic and syngeneic transplants, CD4-SPION-labeled Tregs delivered intravenously produced T2 reduction detectable up to 72 h post-infusion in both groups, compared to untreated controls. Treg treatment maintained the macroscopic integrity of transplanted tissue up to 7 days post-transplantation and reduced visible signs of early inflammatory injury, while untreated controls showed early signs of rejection (Ping et al., manuscript in preparation). Together, these studies suggest that clinically compatible labeling strategies capable of supporting both Treg isolation workflows and longitudinal MRI readouts are within reach, though validation in established preclinical transplant models with immunological endpoints remains a necessary next step toward translational application.

Despite this strong biological rationale, dedicated MRI tracking of Tregs in GvHD models remains largely unexplored. Most available imaging data in the GvHD context come from intravital microscopy studies, such as Lin et al.’s work using two-photon imaging to track donor allogeneic effector and regulatory T cells with host dendritic cells during GvHD [121], or bioluminescence [51] or PET [58,122] studies, rather than whole-body MRI. A notable exception is the metabolic imaging approach of Assmann et al., who showed that the glycolytic activity of pathogenic T cells in GvHD could be detected by ^13^C-MRI, demonstrating that MRI-based immune monitoring in this setting is feasible, albeit through a functional readout rather than direct cell tracking [123]. We believe the slow adoption of MRI in this space reflects a practical mismatch between GvHD model biology and current MRI sensitivity constraints: the multi-organ distribution of disease targets (skin, gut, liver) means that any whole-body ^19^F acquisition must compete with a highly diffuse signal that is unlikely to concentrate sufficiently for voxel-level detection. A more tractable initial approach would focus on a single target organ with high expected Treg accumulation. For example, one could image the liver in hepatic GvHD using high-field preclinical scanners to optimize sensitivity before scaling toward whole-body protocols. SPION-based tracking of infused Tregs in the gut is also feasible but would require careful experimental controls to distinguish labeled Treg signal from the iron-rich intestinal background and macrophage-mediated confounders discussed in Section 3.2. Prioritizing these focused, organ-specific studies would establish whether MRI biomarkers can predict treatment response in GvHD before committing to more ambitious whole-body protocols.

### 4.2. Autoimmune Diseases

MRI-based tracking of Tregs and other immunomodulatory cells in autoimmune disease models is among the most developed applications in the field, with diabetes T cell trafficking studies establishing the methodological groundwork for subsequent Treg imaging work. The landmark study by Srinivas et al. established ^19^F MRI as a quantitative tool for in vivo T cell tracking in a model of autoimmunity [104]. Using the NOD mouse model of type 1 diabetes, they tracked fluorine-labeled CD4^+^ T cells after adoptive transfer. Approximately 2% of the cells were detected in the pancreas at 48 h, and quantification from the ^19^F images correlated with fluorescence microscopy and ^19^F spectroscopy of excised pancreas. More importantly, the labeling procedure did not affect T cell migration, proliferation, or function. This study established several features that remain important benchmarks: background-free detection, quantification capability, and validation against orthogonal methods.

**Figure 5:**
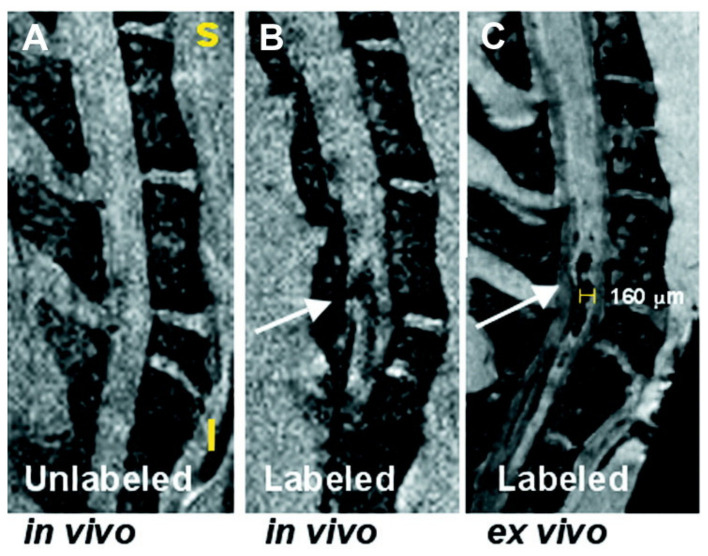
In vivo and ex vivo MRI detection of iron-labeled encephalitogenic T cells in the spinal cord of EAE mice. (**A**) In vivo T2*-weighted MRI of unlabeled T cell-receiving mice shows no focal hypointense lesions in the thoracolumbar spinal cord. (**B**) EAE mice that received iron-labeled T cells show distinct hypointense lesions in the spinal cord, indicating specific T cell homing to the spinal cord. (**C**) Ex vivo magnetic resonance microscopy of the same labeled mouse confirms the lesion. Adapted with permission from Ref [124]. Copyright © 2004 John Wiley & Sons, Inc.

In the experimental autoimmune encephalomyelitis (EAE) model of multiple sclerosis [124], SPION-labeled T cells have been tracked crossing the blood–brain barrier, providing insights into the spatiotemporal dynamics of CNS T cell infiltration that would be inaccessible by ex vivo methods (Figure 5). Baeten et al. demonstrated the feasibility of tracking myelin-reactive T cells labeled with SPIO particles in the CNS of EAE rats using MRI [125]. Upon adoptive transfer, labeled T cells were detected in the sacral spinal cord in naïve recipients, but showed broader CNS distribution, including the brain, in pre-immunized animals, illustrating how host immune context shapes trafficking patterns. Wuerfel et al. complemented this work by optimizing a VSOP-based labeling protocol for MRI tracking of encephalitogenic T cells in EAE [126].

Extending ^19^F MRI to tolerogenic cell types, Cooke et al. demonstrated successful fluorine labeling of therapeutic human tolerogenic dendritic cells (Tol-DCs) with the commercial agent Cell Sense [127]. Labeling did not affect cell viability or immunosuppressive function. Labeled Tol-DCs were detectable by clinical-field MRI in a murine model of rheumatoid arthritis, providing a direct precedent for applying similar workflows to Tregs in autoimmune settings. These studies collectively establish that both iron oxide and ^19^F MRI approaches can resolve T cell or Treg trafficking patterns in autoimmune tissues with sufficient sensitivity for preclinical studies, and they define the technical groundwork for future Treg-specific tracking in type 1 diabetes, multiple sclerosis, and rheumatoid arthritis models for which Treg ACT is under active investigation.

## 5. Treg Tracking in the Clinical Space: Hopes for the Future

### 5.1. Clinical MRI Cell Tracking Studies

Despite hundreds clinical trials investigating Tregs as therapy [50] and thousands examining T cell therapy broadly [128], the clinical translation of MRI-based cell tracking has advanced incrementally, with early studies using SPION-labeled dendritic cells to establish the safety and feasibility of labeled cell infusion in cancer patients [129]. These foundational studies [129,130,131,132], reviewed in the context of four early clinical trials by Bulte and colleagues [66,133], confirmed that SPION-labeled cells can be administered safely, detected in vivo by clinical MRI, and localized to relevant anatomical compartments.

**Figure 6:**
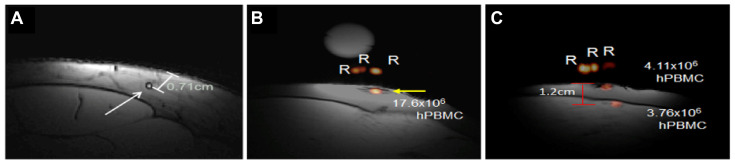
MRI detection of ^19^F-labeled human PBMCs in a clinically relevant model at 3T. (**A**) Representative proton image of a human leg obtained with the same orientation and imaging parameters demonstrates tissue appearance and subcutaneous fat deposition similar to those found in the ham shank model shown in (**B**,**C**); the arrow marks a lymph node which serves as injection depth calibration for the ham shank, located at an approximate depth of 0.71 cm. (**B**) ^19^F MRI demonstrates detectable signal following intradermal injection of 17.6 × 10^6^ labeled human PBMC in the ham shank. (**C**) ^19^F MRI also detects lower administered cell doses, including 4.11 × 10^6^ labeled human PBMCs injected intradermally and 3.76 × 10^6^ labeled human PBMCs injected intramuscularly, with the deeper signal visualized at approximately 1.2 cm below the surface. R, reference markers. PBMCs, peripheral blood mononuclear cells. Adapted from [134] under a Creative Commons Attribution 4.0 International License.

A significant step towards Treg-related clinical use came from Fink et al. [134]. They showed that human peripheral blood mononuclear cells (PBMCs) labeled with a ^19^F perfluorocarbon agent under GMP-compliant conditions could be detected in living animals and in ex vivo human-like models using clinical MRI protocols (Figure 6). More than 99% of PBMCs were successfully labeled without compromising their functionality or viability. Researchers detected labeled PBMCs at the injection site and in a draining lymph node in a mouse model. They also optimized a clinical cellular MR protocol to detect labeled cells at depths comparable to a human lymph node using a dual ^1^H/^19^F radiofrequency coil. This work proved that GMP-compliant ^19^F cell labeling and clinical-field MRI detection are technically feasible for human immune cell products.

The first direct clinical proof of ^19^F MRI tracking of adoptively transferred T cells in a human patient was reported by Ahrens and team [135]. In a pilot study involving a patient with relapsed, refractory HPV-mediated head and neck squamous cell carcinoma, researchers expanded autologous TILs from a resected lung metastasis, labeled them with a PFC nanoemulsion tracer, and infused about 7 × 10^10^ cells labeled in a single GMP-compatible batch while ensuring more than 90% viability and standard release criteria. At 22 days after infusion, quantitative single-voxel ^19^F MRS of the liver at 3T estimated that around 30% of transferred TILs had died off, taking advantage of how hepatic Kupffer cells clear PFC released from dying cells. While this method measured cell survival and not anatomical movement, it provided a quantitative in vivo assessment of T cell fate after adoptive transfer that cannot be obtained from peripheral blood alone. This sets a direct conceptual example for Treg tracking applications. Likewise, O’Hanlon et al. showed that the ^19^F tracer Cell Sense can be incorporated into the clinical-scale manufacturing process of a T cell immunotherapy product without affecting the final product or its cryopreservation [113]. These manufacturing demonstrations are crucial because they confirm that MRI tracking can be integrated into the production process rather than being an added step after manufacturing, allowing for tracking without further manipulation of the cells.

### 5.2. Active Clinical Trials

The clinical development of MRI-based immune cell tracking is now entering an active phase, with registered trials moving the technology from preclinical validation into human subjects for the first time. NCT02921373, led by Dekaban and colleagues, is a first-in-human feasibility study testing ^19^F Cell Sense-labeled autologous PBMCs administered intradermally to healthy adults and prostate cancer patients. The primary objectives are to confirm and optimize ^19^F/^1^H dual-coil detection parameters at a clinical field strength, and to determine whether labeled PBMC migration to a draining lymph node can be detected in vivo. The cell dose (3 × 10^6^ labeled PBMCs per subject) is intentionally conservative, designed to establish a safety and detectability baseline rather than a therapeutic effect. This trial directly confronts the sensitivity question that preclinical and ex vivo tissue studies cannot answer: whether the ^19^F signal from a clinically realistic immune cell product survives the transition to a living human subject with sufficient contrast for anatomical localization. NCT07075523 represents a more recent registration investigating MRI-based T cell tracking in an immunotherapy context. Although registered in 2024, full protocol details are still emerging.

Taken together, these trials mark a genuine inflection point. The field is no longer asking whether MRI cell tracking is theoretically translatable, but whether it works under the constraints of clinical practice. The path from these studies to Treg-specific tracking in transplant or autoimmune settings will require three additional demonstrations: that sensitivity is adequate at the cell doses used in Treg therapy, that labeling agents carry acceptable safety profiles in immunologically vulnerable patient populations, and that labeled Treg products satisfy regulatory release criteria without compromising the manufacturing workflow.

### 5.3. Barriers to Clinical Translation and Future Directions

Despite the technical progress noted above, several challenges must be overcome before MRI-based Treg tracking becomes standard in clinical trials, and not all barriers carry equal weight. We argue that sensitivity is the rate-limiting bottleneck. Treg therapy trials generally use infusion doses of 10^8^ to 10^9^ cells [107], and these cells distribute throughout the body [136]. The minimum detectable amount for ^19^F MRI is about 10^6^ cells per voxel at clinical field strengths [13,134], meaning that Treg accumulation in organs might only be detectable when cells concentrate in specific areas. Improvements in perfluorocarbon formulation with higher fluorine density, along with advancements in ^19^F coil design and pulse sequence optimization, are key research areas to enhance sensitivity and clinical translation potential. SPION-based approaches offer higher sensitivity in principle, but as argued in Section 3.2, this advantage is largely negated by specificity problems in the inflamed microenvironments where Treg tracking is most clinically meaningful.

The regulatory pathway, by contrast, may be more tractable than it appears. Both iron oxide and ^19^F labeling methods have shown GMP compatibility in human T cell products, and the CD25/CD4 microbead labeling strategy is particularly promising because it integrates into the magnetic-assisted cell sorting isolation step already used in many Treg manufacturing protocols. Kiru et al.’s mechanoporation technique represents another GMP-scalable method specifically validated for CAR-T cells [80]. Regardless of the approach, any labeling agent included in a clinical product generally requires an investigational new drug (IND) exemption or approval from the relevant regulatory authority [66,137]. The FDA has not approved any imaging agent specifically for MRI-based cell tracking. The closest approved options are Miltenyi’s CD34 microbead system, which has FDA humanitarian use device approval [85] for treating acute myeloid leukemia, and Ferumoxytol, an iron supplement used in the treatment of anemia [138]. The CD25/CD4 microbeads used for Treg isolation belong to the same reagent class, which provides a potential basis for an “off-label” imaging application that could follow a similar regulatory path. The critical point is that the regulatory hurdles, while real, are not novel. They follow established frameworks. The scientific hurdles of sensitivity and functional validation, discussed above and in Section 3.4, are where investment is most needed.

The main argument for adding MRI tracking to clinical Treg trials is that imaging results can generate pharmacodynamic information that blood counts cannot. If graft-site Treg accumulation measured by MRI correlates with reduced rejection rates or decreased immunosuppression burden, it becomes a biomarker capable of informing dose, route, and timing decisions in ways that transform trial design. Orozco et al. have highlighted exactly these unanswered questions for SOT Treg therapy [136]. Achieving that correlation will require prospective studies that pair imaging endpoints with immunological and clinical outcomes, a standard that the current preclinical literature has not yet met. Establishing this link in one well-powered preclinical transplant or autoimmune model, using a GMP-compatible labeling platform and validated functional release criteria, would represent the most valuable single contribution the field could make toward clinical translation of MRI Treg tracking.

The progress made in preclinical studies, including SOT, GvHD, and autoimmune models, combined with the developing clinical framework for labeled Treg cell tracking, positions the field well for initial human Treg tracking studies in the next decade. However, no current imaging strategy fully captures the complete in vivo fate of immune cell therapies, including both biodistribution and functional status. As the field advances, future cell tracking approaches will need to integrate multimodal approaches to better determine not only where therapeutic Tregs localize, but also whether they remain viable, stable, and immunosuppressive after infusion. Key milestones will involve standardizing labeling protocols, developing clinical-field sensitivity and functionality benchmarks, and proving imaging outcome relationships in prospective preclinical studies and early phase clinical trials.

## Figures and Tables

**Table 1:** Comparison of in vivo T cell tracking methods. Comparisons are based on typical performance in a cellular imaging context. Values represent typical ranges reported across heterogeneous experimental conditions, including different contrast agents, field strengths, scanner platforms, and T cell types. Compatibility levels (High, Moderate, Low, Low-moderate, None, Developing) express the feasibility of such image modality in tracking T cells in vivo. Readiness (Ready, Preclinical, Developing) expresses the feasibility of imaging modality in application to clinical use. Abbreviations: MRI = Magnetic Resonance Imaging; PET = Positron Emission Tomography; SPECT = Single-Photon Emission Computed Tomography; MPI = Magnetic Particle Imaging; PAI = Photoacoustic Imaging; mm = millimeter; µm = micrometer; cm = centimeter; M = molar.

Method	Sensitivity with Contrast Agents	Spatial Resolution	Tissue Penetration	Longitudinal Capabilities	Detection Limit (Cells)	Clinical Translation Readiness
**MRI**	10^−3^ to 10^−5^ M [15]	0.1–1 mm [15]	No limit [15]	High [15]	10^3^ to 10^5^ [60]	Ready [15]
**Optical imaging**	10^−9^ to 10^−12^ M [15]	2–5 mm (whole-body imaging) [15]	Poor (<2 cm) [15]	High [15]	10^3^ to 10^5^ [61]	Preclinical [15]
**PET/SPECT**	10^−10^ to 10^−12^ M [15]	5–10 mm [15]/0.3–15 mm [62,63]	No limit [15]	Moderate [15]	10^4^ to 10^5^ [64]	Ready [15]
**Ultrasound**	10^0^ M [15]	1 mm [15]	Moderate (cannot pass bone/air) [15]	High [15]	Contrast-agent dependent [65,66,67]	Preclinical [15]
**MPI**	10^−6^ to 10^−8^ M [68]	0.5–2 mm [69]	No limit [68,69]	High [70]	10^2^ to 10^4^ [71]	Developing [69]
**PAI**	10^−10^ to 10^−12^ M [72]	10–500 µm [73]	Moderate (mm-cm) [73]	High [56]	10^3^ to 10^5^ [56]	Developing [73]

## Data Availability

No new data were created or analyzed in this study. Data sharing is not applicable to this article.

## Abbreviations

The following abbreviations are used in this manuscript:

^1^H	proton
^9^F	fluorine-19
ACT	adoptive cell therapy
APC	antigen-presenting cell
CAR	chimeric antigen receptor
CNS	central nervous system
DAMPs	damage-associated molecular patterns
dd-cf	donor-derived cell-free
EAE	experimental autoimmune encephalomyelitis
FDA	Food and Drug Administration
F68	block co-polymer F68
FOXP3	forkhead box P3
GMP	good manufacturing practice
GvHD	graft-versus-host disease
HIV-tat	human immunodeficiency virus trans-activator of transcription
HLA	human leukocyte antigen
HSV-TK	herpes simplex virus thymidine kinase
IL-2	interleukin-2
IL-10	interleukin-10
IND	investigational new drug
IOPC-NH2	superparamagnetic nano-sized iron oxide particle
MHC	major histocompatibility complex
MIRB	Molday Ion Rhodamine-B
MPI	magnetic particle imaging
MRI	magnetic resonance imaging
MRS	magnetic resonance spectroscopy
NOD	non-obese diabetic
PAI	photoacoustic imaging
PAMP	pathogen-associated molecular pattern
PAT	photoacoustic tomography
PBMC	peripheral blood mononuclear cell
PFC	perfluorocarbon
PET	positron emission tomography
PFCE	perfluoro-15-crown-5-ether
PFPE	perfluoropolyether
PSMA	prostate-specific membrane antigen
QC	quality control
SOT	solid organ transplant/solid organ transplantation
SPECT	single-photon emission computed tomography
SPIO/SPION	superparamagnetic iron oxide/nanoparticle
TCR	T cell receptor
TGF-β	transforming growth factor beta
Th17	T helper 17
TIL	tumor-infiltrating lymphocyte
Tol-DCs	tolerogenic dendritic cells
TRuC	T cell receptor fusion construct
Treg	regulatory T cell
USPIO	ultrasmall superparamagnetic iron oxide
VSOP	very small superparamagnetic iron oxide particle
